# Cell type-dependent HIF1 α-mediated effects of hypoxia on proliferation, migration and metastatic potential of human tumor cells

**DOI:** 10.18632/oncotarget.17806

**Published:** 2017-05-11

**Authors:** Enikő Tátrai, Alexandra Bartal, Alexandra Gacs, Sándor Paku, István Kenessey, Tamás Garay, Balázs Hegedűs, Eszter Molnár, Mihály T. Cserepes, Zita Hegedűs, Nóra Kucsma, Gergely Szakács, József Tóvári

**Affiliations:** ^1^ Department of Experimental Pharmacology, National Institute of Oncology, Budapest, Hungary; ^2^ Central Pharmacy, National Institute of Oncology, Budapest, Hungary; ^3^ 1st Institute of Pathology and Experimental Cancer Research, Semmelweis University, Budapest, Hungary; ^4^ Tumor Progression Research Group, Hungarian Academy of Sciences, Semmelweis University, Budapest, Hungary; ^5^ 2nd Department of Pathology, Semmelweis University, Budapest, Hungary; ^6^ National Cancer Registry, National Institute of Oncology, Budapest, Hungary; ^7^ Department of Thoracic Surgery, Medical University of Vienna, Vienna, Austria; ^8^ Department of Thoracic Surgery, Ruhrlandklinik, University Duisburg-Essen, Essen, Germany; ^9^ Institute of Enzymology, Research Center for Natural Sciences, Hungarian Academy of Sciences, Budapest, Hungary; ^10^ Semmelweis University, Budapest, Hungary

**Keywords:** tumor cell motility, hypoxia, small GTPases, RhoA activation, metastasis

## Abstract

Tumor hypoxia promotes neoangiogenesis and contributes to the radio- and chemotherapy resistant and aggressive phenotype of cancer cells. However, the migratory response of tumor cells and the role of small GTPases regulating the organization of cytoskeleton under hypoxic conditions have yet to be established. Accordingly, we measured the proliferation, migration, RhoA activation, the mRNA and protein levels of hypoxia inducible factor-1alpha (HIF-1α) and three small G-proteins, Rac1, cdc42 and RhoA in a panel of five human tumor cell lines under normoxic and hypoxic conditions. Importantly, HT168-M1 human melanoma cells with high baseline migration capacity showed increased HIF-1α and small GTPases expression, RhoA activation and migration under hypoxia. These activities were blocked by anti- HIF-1α shRNA. Moreover, the *in vivo* metastatic potential was promoted by hypoxia mimicking CoCl_2_ treatment and reduced upon inhibition of HIF-1α in a spleen to liver colonization experiment. In contrast, HT29 human colon cancer cells with low migration capacity showed limited response to *in vitro* hypoxia. The expression of the small G-proteins decreased both at mRNA and protein levels and the RhoA activation was reduced. Nevertheless, the number of lung or liver metastatic colonies disseminating from orthotopic HT29 grafts did not change upon CoCl_2_ or chetomin treatment. Our data demonstrates that the hypoxic environment induces cell-type dependent changes in the levels and activation of small GTPases and results in varying migratory and metastasis promoting responses in different human tumor cell lines.

## INTRODUCTION

Oxygen supply is essential for the growth of cells and is often reduced in solid tumors, particularly in the center of the tumor mass. Adaptation to the hypoxic condition supports tumor growth and survival. Furthermore, tumor hypoxia might be associated with resistance to radio- and chemotherapy [1–3]. HIF-1 (hypoxia inducible factor-1), the major regulator of cellular response to tissue hypoxia, is a heterodimer protein consisting of HIF-1α and HIF-1β subunits [4]. Under normal oxygen tension HIF-1α binds to the von Hippel-Lindau protein and is degraded via the ubiquitin-dependent pathway [5]. During this process, specific proline residues undergo posttranslational hydroxylation by prolyl hydroxylases [6]. Under hypoxia, HIF-1α heterodimerizes with the constitutively produced HIF-1β subunit within the nucleus and binds to the hypoxia responsive element (HRE). In this way HIF-1 activates different genes involved in angiogenesis, migration and survival [7], such as erythropoietin or VEGFs [8]. The same genes are often found to be overexpressed in tumor cells, suggesting that these pathways are also involved in tumor progression [9].

The members of Rho family small G-proteins, Rac1, cdc42 and RhoA are approximately 21 kDa proteins that regulate a wide spectrum of cellular functions, such as cell growth, cytoskeletal reorganization, membrane trafficking and angiogenesis [10]. Their activity is regulated by guanine nucleotide exchange factors (GEFs), guanine nucleotide dissociation inhibitors (GDIs) and GTPase-activated proteins (GAPs) [11]. Increasing amount of evidence supports the role of Rho GTPases in tumorigenesis [12], metastasis formation [13], cell-cycle control [14] and apoptosis [15]. HIF-1α has been described to influence cell motility via the regulation of the expression and activation of RhoA. In a mesenchymal stem cell migration model the amount of active RhoA was reduced at 1% oxygen level, whereas activation of RhoA under hypoxic conditions rescued migration [16]. In a recent publication, Gilkes et al described 3 direct HIF binding sites on RhoA and ROCK1 genes as well, indicating the HIF-mediated regulation of the expression of these proteins. In their breast cancer model, hypoxia enhanced cell migration through actin polymerization, myosin light chain kinase phosphorylation and activation of focal adhesion kinase [17].

Although hypoxic conditions have been shown to increase tumor cell motility [18, 19] and aggressiveness [20] through the activation of small GTPases, the cell-type dependence of the expression and activation of RhoA, cdc42 or Rac1 is unknown. Therefore, the aim of this study was to investigate the effect of hypoxia on the expression of HIF-1α, RhoA, cdc42 and Rac1, RhoA activation, cell proliferation and migration using five tumor cell lines of different tissue origin. Additionally, the *in vivo* metastatic potential was measured in the different model system using CoCl_2_ treatment for the stabilization of HIF-1α.

## RESULTS

### Differential proliferative response to experimental hypoxia

Hypoxia had a cell-type dependent effect on tumor cell proliferation. However, the increased proliferation of HT1080 human fibrosarcoma cells at 5% O_2_ level was the only statistically significant alteration. In contrast, 1% O_2_ level modestly decreased the proliferation of HT1080 cells. While the proliferation capacity of the HT168-M1 melanoma and HT25 colon carcinoma cell lines modestly increased both at 1% and 5% O_2_ levels compared to normoxia, the proliferation of HT29 colon carcinoma cells decreased, especially at the 1% O_2_ level. No differences were detected between hypoxic and normoxic PE/CA PJ15 head and neck carcinoma cells (Table 1).

**Table 1:** Effect of hypoxia on the proliferation of different tumor cells

	5% O_2_	1% O_2_
HT168-M1	121±4.9	110±1.34
HT1080	154±16.9*	65±16.6
HT25	126±8.4	107±5.6
HT29	88±3.2	66±16.9
PE/CA PJ15	107±2.7	106±5.3

Data are means±SD in percentage of control (normoxic) values under different hypoxic conditions. Representative data are from at least 6 parallels from three independent investigations; *p<0.05.

### Varying effect of hypoxia on tumor cell migration

**Figure 1:**
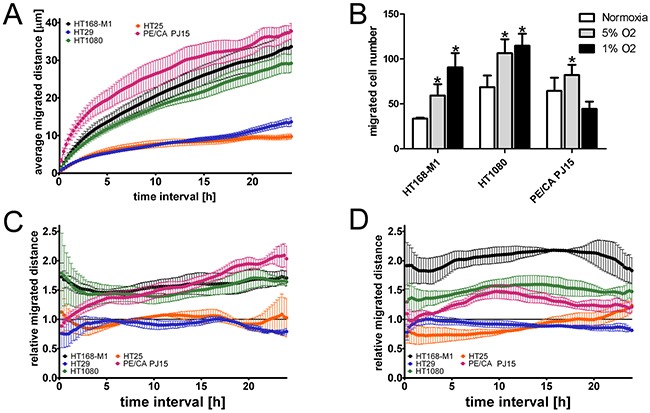
Effect of hypoxia on tumor cell migration **(A)** Baseline motility of the five tumor cell lines measured under normoxic conditions for 24 h using time-lapse videomicroscopy. Curves represent the mean ± SD of migrated distance during the test period. Note that the two colon carcinoma cell lines showed very low migration capacity. **(B)** Migration of three tumor cell lines in Boyden chamber using fibronectin as chemoattractant. Cells were preincubated under hypoxic conditions for 72 h prior to the 6 h (HT168-M1, HT1080) or 20 h (PE/CA-PJ15) migration test at normoxia. Cell migration was quantified by counting the cells in the lower chambers covered with the chemoattractant fibronectin. Data are mean ± SD, n=6; *p<0.05. In the time-lapse videomicroscopic test the relative migration capacity of the cells was determined based on the analysis of snapshots of microscopic fields taken every 5 minutes for 1 day at normoxic conditions and for 3 days at 5% **(C)** or 1% **(D)** oxygen levels. The captured phase contrast pictures were analyzed using a cell-tracking program enabling manual marking of individual cells. Relative migration distance is represented by the ratio of the average displacement of the cells under hypoxia and normoxia (mean ± SD). 1= same migration capacity of the cells under hypoxic and normoxic condition.

Next, we characterized the effect of hypoxia on the motility of the cells. First we used time-lapse videomicroscopy to measure the baseline migration capacity of the five tumor cell lines. HT168-M1, HT1080 and PE/CA PJ15 cells migrated at a relatively high speed, while the two colon carcinoma cell lines (HT25 and HT29) were moving very slowly (Figure 1A). To measure the effect of hypoxia on fibronectin-induced migration cells were analyzed in Boyden chamber assay. Tumor cells were allowed to migrate for 6 or 20 hours, following a 72-hour preincubation period at 1% and 5% O_2_ concentration or under normoxic conditions. Preincubation under hypoxic conditions resulted in increased migration of HT168-M1 and HT1080 cells. In the case of PE/CA PJ15 cells only the 5% O_2_ level increased significantly the migration compared to normoxic condition (Figure 1B). The HT25 and HT29 cell lines displayed no migration capacity in the Boyden chamber assay, even under normoxic conditions. Thus we further investigated the effect of hypoxia using time-lapse videomicroscopy. Cells were monitored under normoxic conditions for 24 h and then the oxygen tension was changed to 5% or 1% for 72 hours. Figure 1C-1D show the migration of the cells detected during the 72-96 h period (third day in hypoxia) relative to that observed during the first 24 hours (normoxia). The results of the time-lapse videomicroscopy and Boyden chamber migration assay showed a good correlation. HT1080 and HT168-M1 cells showed increased migration under both hypoxic conditions, and HT168-M1 cells moved faster as oxygen tension decreased. PE/CA PJ15 cells showed increased motility at 5% O_2_ level, but there were no significant changes under 1% O_2_ hypoxia compared to normoxic control. The motility of the two colon cancer cell lines (HT25 and HT29) was unchanged under hypoxia compared to normoxic condition.

Although, due to the technical setting of the experiment, the results of the time-lapse video microscopy exclude the possible effect of reoxygenization in the induction of cell movement under hypoxia, we repeated the Boyden chamber experiment without preincubation of the cells in hypoxic conditions. HT168-M1 tumor cells from normoxic conditions were allowed to migrate in hypoxic chamber for 6 hours and cell migration was found increased in this experimental setup as well (number of migrating cells per field of view was 44 ± 8.9 cells in normoxia and 86 ± 6.4 cells in hypoxia).

### Cell-type dependent changes in expression of HIF-1α and cytoskeleton regulating small G-proteins in hypoxia

**Figure 2:**
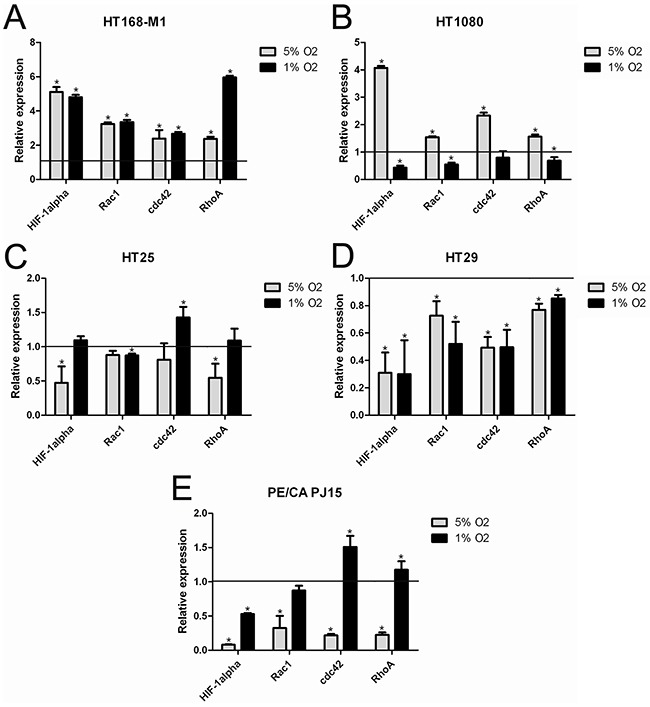
mRNA expression of HIF-1α, RhoA, Rac1 and cdc42 under normoxic and hypoxic conditions **(A-E)** Relative mRNA expression under hypoxic conditions (72h incubation) compared to normoxia (normoxic values=1). Data are mean ± SD, n=3, *p<0.05.

To characterize the molecular background of the response to hypoxia, we examined the mRNA levels of HIF-1α, Rac1, cdc42 and RhoA in the different cell lines after 72h hypoxia treatment. In the case of HT168-M1 all of the examined markers were increased at both 5% and 1% O_2_ level (Figure 2A). In HT1080 cells, HIF-1α mRNA expression increased 4-fold in 5% O_2_ environment and the other markers were also higher compared to the normoxic values (Figure 2B). However, mRNA levels remained normal or decreased at 1% O_2_.

Interestingly, HIF-1α mRNA expression in HT25 cells decreased only at 5% O_2_ level (Figure 2C), while in HT29 (Figure 2D) and PE/CA PJ15 (Figure 2E) in both (5% and 1% O_2_) hypoxic conditions. HT29 cells demonstrated decreased expression of all examined small G-proteins RNA at both hypoxic oxygen levels.

### Differential hypoxia induced RhoA activation in tumor cells

We used an ELISA-based method for the detection of active RhoA following a 72 h preincubation of the cells under hypoxic conditions. In HT168-M1 cells the level of active RhoA was increased under both hypoxic conditions. Similar results were found in the case of HT1080 cells at 5% O_2_ level. However, in HT29 cells the activation of RhoA significantly decreased at both reduced oxygen levels. In HT25 and PE/CA PJ15 cells no significant changes in RhoA activation were found. (Table 2).

**Table 2:** RhoA activation in the different cell lines under hypoxic conditions

	5% O_2_	1% O_2_
HT168-M1	**150.5 ± 1.9** *	**130.8 ± 1.0** *
HT1080	**171.7 ± 10.2** *	100.3 ± 0.4
HT25	119.8 ± 21.2	95.1 ± 9.5
HT29	**77.4 ± 4.3** *	**75.8 ± 7.8** *
PE/CA PJ15	104.8 ± 1.6	112 ± 5.7

Data are means ± SD in percentage of control (normoxic) values under different hypoxic conditions; representative data of 3 independent experiments. *p<0.05

### The effect of hypoxia on protein expression of the three small GTPases and HIF-1α

**Figure 3:**
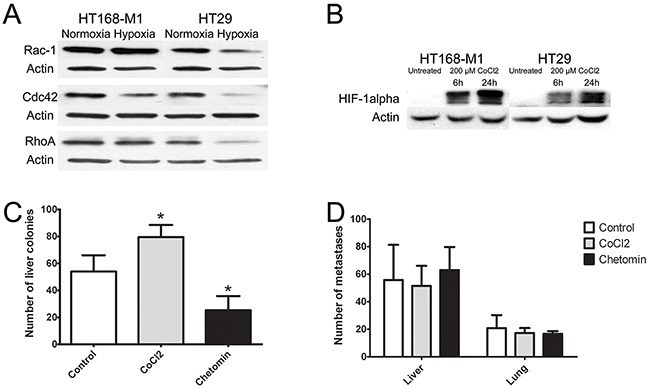
Effect of hypoxia on protein expression and *in vivo* metastasis of human tumor xenografts **(A-B)** Protein expression of small GTPases and HIF-1α in HT168-M1 and HT29 cells under hypoxic conditions (1% O_2_ level) compared to normoxia (a representative blot). **(C)** HT168-M1 human melanoma cells were injected intrasplenically and liver colonies formed were counted at day 34 of the treatment. **(D)** HT29 human colon cancer fragments were orthotopically transplanted to the appendix region and liver and lung metastases were counted at day 34. Note that the CoCl_2_ treatment increased metastasis formation by the highly motile HT168-M1 cells. Inhibition of HIF proteins by chetomin, on the other hand, resulted in a significant decrease in the metastatic potential. However, CoCl_2_ or chetomin treatment had no effect on the metastatic capacity of HT29 cells. Data represent mean ± SEM of two independent experiments; *p<0.05.

For the further analysis of the effect of hypoxic environment on tumor cells we selected a cell line with high baseline migration capacity (HT168-M1) and one with low migration capacity (HT29). In these cells, protein expression level of RhoA, Rac1 and cdc42 was measured by Western blot analyses. Most of the results correlated with findings at the mRNA level: RhoA and Rac1 protein levels were increased in HT168-M1 cells, but all the three proteins were decreased in HT29 cells after 72 h incubation in hypoxia (1% O_2_ level, Figure 3A). Because we used CoCl_2_ for the stabilization of HIF-1α in our animal experiments, the HIF-1α protein levels were measured in CoCl_2_-treated cells as well to control HIF-1α induction. HIF-1α protein level was increased after hypoxia induction by 6 and 24 h CoCl_2_ treatment in both cell lines (Figure 3B).

### Experimental hypoxia changes metastatic potential in a cell-type dependent manner

*In vivo* hypoxia was generated by adding CoCl_2_ to the drinking water (260 mg/l during the whole period of the experiment). HIF-1α activity was blocked by chetomin treatment (1 mg/kg dissolved in DMSO *i.p*., twice a week for 4 weeks). The effect of hypoxia was evaluated in colonization and metastasis models.

In the xenograft model of highly migratory HT168-M1 cells the number of metastases was significantly higher in the CoCl_2_ group, and was lower in the chetomin-treated group compared to the untreated controls (Figure 3C). However, in the HT29 (cells with limited baseline migration activity) metastasis model there were no significant changes in the number of lung or liver metastases of the CoCl_2_-treated group compared to the controls (Figure 3D).

### HIF-1α downregulation changes hypoxia-induced small G-protein expression and RhoA activation

**Figure 4:**
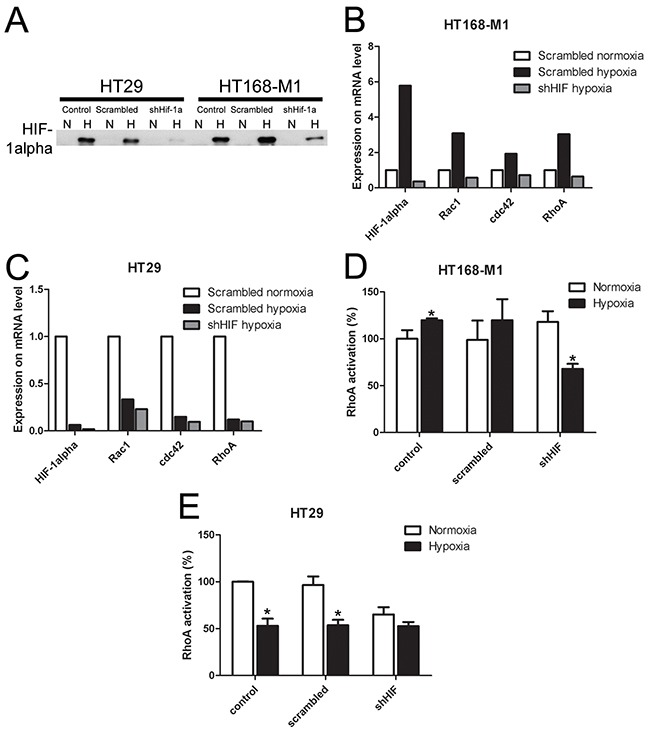
Effect of HIF-1α gene silencing on protein and mRNA expression and RhoA activation **(A)** HIF-1α protein expression in HIF-1α-silenced HT168-M1 cell lines. N: normoxic, H: hypoxic (1% O_2_) conditions. **(B-C)** Relative mRNA expression under hypoxic conditions compared to normoxic scramble-transfected human tumor cells (normoxic values=1, representative figures). **(D-E)** RhoA activation in control, scramble-treated and HIF-1α silenced tumor cells under hypoxic (1% O_2_) conditions. Data are mean ± SD, n=3, *p<0.05.

In order to demonstrate that HIF-1α mediates the hypoxia-induced changes in small G-protein expression, migration and metastasis formation, we decreased HIF-1α level by shRNA method in the two selected cell lines. The shRNA significantly decreased HIF-1α protein expression in both investigated cell lines under hypoxic (1% O_2_) condition (Figure 4A). The increased gene expression of HIF-1α and the three small G-proteins under hypoxic conditions (1% O_2_ level), described above, was blocked by shRNA treatment in HT168-M1 cells (Figure 4B). The shRNA treatment had no effect on the decreased gene expression in hypoxia in HT29 cells (Figure 4C). Moreover, HIF-1α silencing in HT168-M1 cells did not change RhoA activation in normoxia, but blocked the induction of RhoA activation under hypoxic (1% O_2_ level) conditions (Figure 4D). At the same time, in HIF-1α silenced HT29 cells RhoA activation was decreased under both normoxic and hypoxic conditions (Figure 4E).

### HIF-1α downregulation changes cell migration and metastatic potential

**Figure 5:**
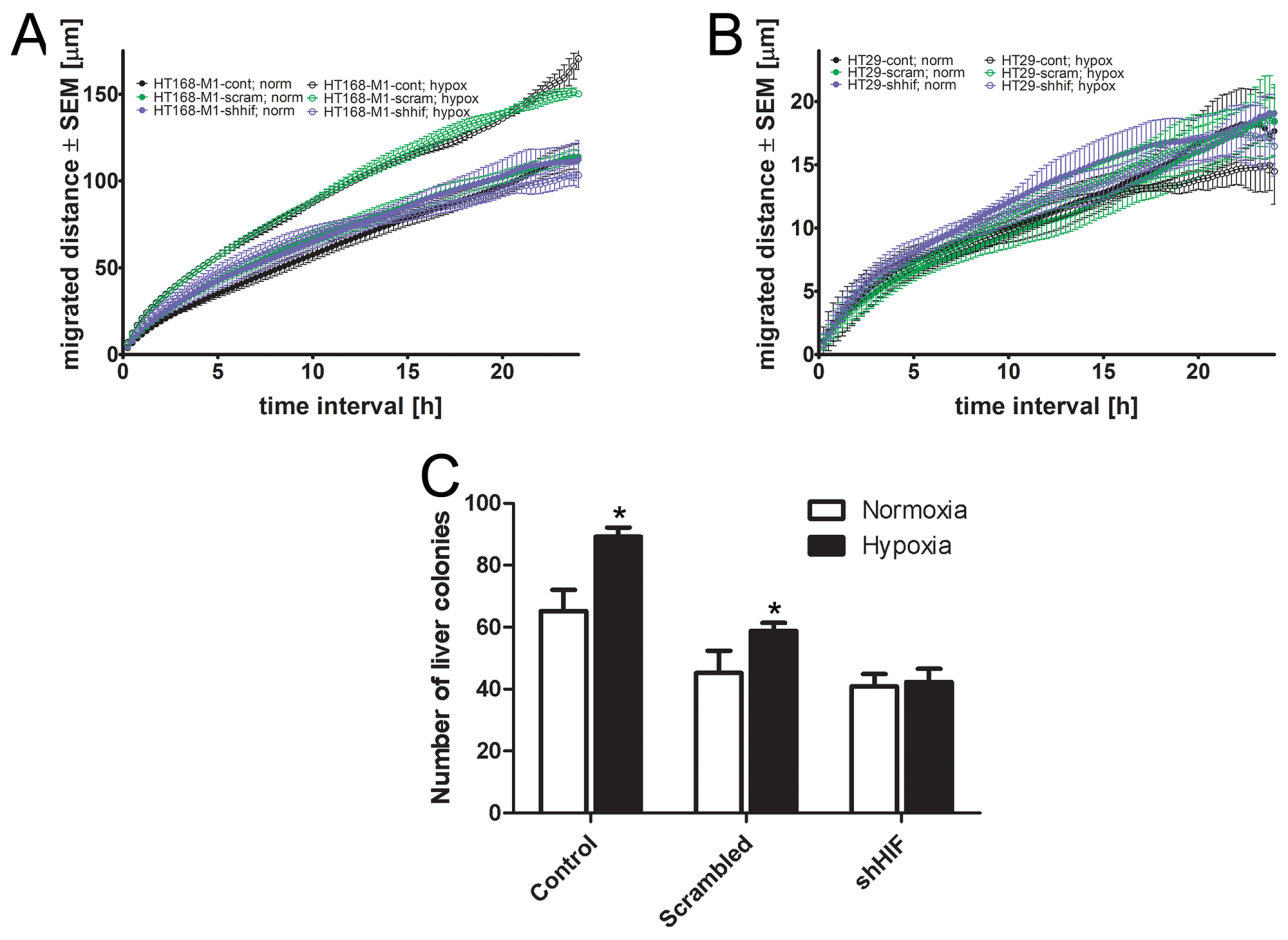
Effect of HIF-1α gene silencing on the migration and *in vivo* metastatic potential **(A-B)**, Baseline motility of HT168-M1 and HT29 tumor cell lines measured under normoxic (24 h) and hypoxic (1% O_2_) conditions for 72 h using time-lapse videomicroscopy. Curves represent the mean ± SD of migrated distance during the test period. **(C)**, HIF-1α silenced HT168-M1 cells were injected intrasplenically and liver colonies formed were counted at day 34 of the treatment. Hypoxia means CoCl_2_ treatment. Data represent mean ± SEM, *p<0.05.

HIF-1α silencing did not change the migratory activity of HT168-M1 cells under normoxia. However, the increased cell motility in hypoxia (1% O_2_, 72h incubation time) was completely blocked in the HIF-1α silenced cells (Figure 5A). We have found that HIF-1α downregulation had no effect on the migratory capacity of HT29 cells either in hypoxic or normoxic conditions (Figure 5B).

At the same time, the result of *in vivo* liver colonization assay using HT168-M1 cells showed that HIF-1α silencing decreased the CoCl_2_ administration-induced increase in metastatic potential of HT168-M1 (Figure 5C).

## DISCUSSION

A growing body of evidence underlines the importance of decreased oxygen levels in tumor progression, as well as chemo- and radiotherapy resistance of solid tumors [21, 22]. In the present study we demonstrated that hypoxia (1% and 5% O_2_ levels) exerts cell-type dependent effects on *in vitro* cell proliferation in our panel of tumor cell lines. Our results are in line with several previous studies demonstrating that lower oxygen concentration may decrease [23–25] or has no effect on the proliferation of different tumor cells [26].

Migration, in part regulated by RhoA, Rac1 and cdc42 small G-proteins, has a pivotal role in tumor progression and metastasis formation. The hypothesis that hypoxia promotes tumor cell aggressiveness, including increased motility and invasion capacity of the cancer cells [27–29] is supported by a series of experimental findings [30–32]. On the other hand, several studies indicate that in certain tumor cell types hypoxia does not induce a change in migration capacity, suggesting that the inherent motility of tumor cells may influence the migratory response to hypoxia [26]. Therefore, we first determined the baseline migration capacity of all five cell lines. Using time-lapse videomicroscopy under normoxia, the two colon carcinoma cell lines showed restricted migration capacity, while the other three cell types showed significant cell migration. We measured the change in motility under hypoxic conditions compared to the normoxic values and found that lower oxygen level increased the motility in most of the cell lines with higher baseline migration activity (HT168-M1, HT1080, PE/CA PJ15). However, in the two colon cancer cell lines hypoxia had no effect on cell migration. Moreover, we obtained similar results when we used Boyden chamber migration assay under normoxia following a 72 h hypoxic preincubation period: the migration capacity of HT168-M1, HT1080 and PE/CA PJ15 cells was increased, while both HT25 and HT29 colon carcinoma cell lines were immotile in the modified Boyden chamber.

In the case of Boyden chamber assay, reoxygenization may have a role in the migration process, while the migratory capacity of HT168-M1 cells in 6 hour hypoxia without hypoxic preincubation period showed similar result as with 72 h hypoxic preincubation. In a previous study, hypoxic exposure increased the metastatic ability of HT1080 fibrosarcoma cells in a similar experimental setting. Reoxygenation for 6 to 18 h showed a trend for further increase, but it was not significant, while after 24 h of reoxygenation, the metastatic efficiency started to decline and following 48 h of reoxygenation, it was similar to that of the normoxic control groups [33].

To study the molecular response to hypoxia, we measured HIF-1α, RhoA, Rac1 and cdc42 mRNA levels and the activation of RhoA after preincubation for 72 hours under hypoxic conditions. We again found that hypoxia induced changes in mRNA level in a cell-type dependent manner. In HT168-M1 cell line with high motility all four markers demonstrated higher mRNA level under both hypoxic conditions. However, mRNA expression levels in HT1080 cells were increased at 5%, but decreased at 1% oxygen concentration. These results correlate with the proliferation data, but not with the migration analyses. Nevertheless, all four mRNA levels were decreased in the HT29 cell line under both hypoxic conditions. Moreover, we found decreased expression of HIF-1α in several other cell lines. It has been described that in prolonged hypoxia (12 h, 0.5% O_2_) the stability of HIF-1α mRNA is decreased, and the amount of natural antisense HIF-1α is increased, resulting in decreased HIF-1α mRNA level [34]. During the 72 h incubation period applied in our experiment hypoxia may induce similar effect in certain cell lines.

Gilkes et al previously defined 3 HIF binding sites in RhoA and ROCK1 genes [17]. Our result showed that in HT168-M1 cells in which HIF-1α expression was increased at both oxygen levels, RhoA activation was elevated as well, while in HT29 cells the decreased HIF-1α expression correlated with the reduction of RhoA activation. However, in most of the cell lines, RhoA activation was independent of HIF-1α mRNA levels.

For further analyses, we chose the increased migratory and hypoxia responsive HT168-M1 and the less motile and rather hypoxia irresponsive HT29 cell lines. We investigated the protein expression level of HIF-1α and the three small GTPases after hypoxic incubation at 1% O_2_ tension. HIF-1α protein expression was increased in both cell lines under hypoxia. Corresponding with the mRNA levels, protein expression was decreased in HT29 cells, but RhoA and Rac1 expression increased compared to the normoxic amount in HT168-M1 cells.

The *in vivo* metastasis assays showed that CoCl_2_, applied for induction of HIF-1α stabilization, significantly increased the liver colonization capacity of HT168-M1 cells, while the inhibition of HIF proteins decreased the number of tumor foci in the liver. However, there were no changes in the metastatic potential of HT29 xenografts after either induction of hypoxia or inhibition of HIF-1α.

In order to demonstrate the role of HIF-1α in the regulation of cell migration and metastatic potential, we downregulated HIF-1α by shRNA in the HT168-M1 and HT29 cell lines. In the highly motile HT168-M1 melanoma cells, where hypoxia increased the mRNA and protein expression of the three small G-proteins and increased the RhoA activation, HIF-1α silencing reverted these changes. Moreover, while the migration capacity of HIF-1α silenced HT168-M1 cells was unchanged under normoxia, the hypoxia-induced increased motility was blocked by HIF-1α silencing. This result is in line with previous findings where in glioma cell lines downregulation of HIF-1α expression inhibited migration and invasion capacity of the cells under hypoxic conditions [35]. However, in our experimental setup migration was increased under hypoxic conditions in the well-motile melanoma cell line, and HIF-1α silencing blocked this effect. We found similar effect in the *in vivo* metastasis experiment as well. The liver colonization capacity of melanoma cells, increased by CoCl_2_ administration, was decreased in the case of HIF-1α silenced cells. In a previous study, *in vivo* SW480 cells were injected subcutaneously into nude mice and HIF siRNA was intraperitoneally administered; the tumor volume of the siHIF-1α group was much smaller than that of the negative control and saline-treated groups [36]. While this aforementioned study demonstrated the effect of HIF-silencing on primary tumor growth, our study showed the involvement of HIF-1α in the metastatic process.

The majority of previous studies suggest that hypoxic conditions increase tumor cell aggressiveness through the activation of small GTPases [37, 38]. Similar to these findings we hereby demonstrated that the highly motile HT168-M1 melanoma cell line became more aggressive under hypoxia as manifested by increased migration and *in vivo* metastasis formation. Hypoxia induced the expression of HIF-1α, RhoA, Rac1, and cdc42 at mRNA as well as protein level, and enhanced the activation of RhoA. Nevertheless, opposite effects of hypoxia were observed in the case of the less motile HT29 colon cancer cell line. Altogether our study demonstrates that hypoxia-induced changes in cell migration and metastatic potential are cell-type dependent and are at least in part mediated by HIF-1α.

## METHODS

### Cell lines and tissue culture conditions

HT168-M1 melanoma, HT1080 (ATCCCCL-121) human fibrosarcoma, HT25 (MJ Hendrix, University of Iowa, IA), HT29 (ATCCHTB-38) human colon adenocarcinomas and PE/CA PJ15 (ECACC 96121230) head and neck carcinoma cell lines were tested by STR analysis (AmpFISTR Identifiler Kit, fragment analysis by capillary electrophoresis) within 6 months. The highly metastatic HT168-M1 human melanoma cell line, derived from the A2058 cell line (ATCCCRL-11147), were selected in a liver colonization model [39]. PE/CA PJ15 cells were cultured in Iscove's MEM (Sigma-Aldrich, St. Louis, MO, USA) while the others in RPMI-1640 Medium (Sigma-Aldrich), supplemented with 5-10% fetal bovine serum (Sigma-Aldrich) and antibiotics (Penicillin/streptomycin, 1:100, Sigma-Aldrich) in humidified environment with atmospheric air containing 5% CO_2_.

### Hypoxic cultures

To establish hypoxic conditions, cells were placed in a hypoxic chamber (Billups-Rothenberg, San Diego, CA, USA) flushed with a gas mixture of 5% CO_2_, 90% or 94% N_2_ and 1% or 5% O_2_, respectively. The hypoxic chamber was then placed in a 37°C incubator for 72 hours.

Cells from hypoxic cultures were investigated immediately after incubation (mRNA for PCR and protein extraction for Western blot analysis, activation assay, cell count), or used in Boyden chamber migration assay under normoxia.

CoCl_2_, as a chemical-induced hypoxia agent was used *in vitro* to produce the same effect as hypoxic chamber. HT168-M1 and HT29 cells were treated with 200 μM CoCl_2_ under normoxic conditions for 6 and 24 h to confirm the results obtained using the hypoxia chamber.

### HIF-1α silencing

We used a plasmid encoding short hairpin RNA (shRNA) (Gene ID 3091) with MegaTran1.0 transfection reagent (OriGene, Rockville, MD, USA) to silence HIF-1α expression in HT168-M1 and HT29 cell lines. The transfection procedure was performed according to the manufacturer's instructions. After the transfection, puromycin was added as selection marker.

### Proliferation assays

The effect of hypoxia on cell proliferation capacity was determined by proliferation assays. Briefly, 1-5×10^3^ tumor cells in six parallels were plated onto a 96-well plate and allowed to adhere. The next day, plates were placed into the hypoxic chamber. After a 72-hour incubation period cell density was evaluated by sulforhodamine B (SRB) assay (Sigma-Aldrich). Cell were fixed with 10% trichloroacetic acid for 60 minutes and stained with 0.4% sulforhodamine B for 15 minutes, after which the excess dye was removed by washing repeatedly with 1% acetic acid. The protein-bound dye was dissolved in 10 mM Tris base solution for OD determination at 570 nm using Bio-Rad 550 Microplate Reader (Bio-Rad Laboratories, Berkeley, CA, USA). Results were compared to those obtained with control cells (cultured in normoxia for 72 hours) and expressed as average percentages relative to values obtained in control (normoxic) cultures.

### Real-time reverse transcription PCR

Total RNA was isolated using TRIzol Reagent (Invitrogen, Life Technologies, Carlsbad, CA, USA) according to the manufacturer's protocol. The purity and concentration of RNA were determined using a spectrophotometer at 260 nm (NanoDrop, Wilmington, DE, USA). RNA was reverse transcribed using the High-Capacity cDNA Reverse Transcription Kit (Invitrogen, Life Technologies) according to the manufacturer`s instructions. After reverse transcription, cDNA samples were stored at −70°C until further processing. Quantitative real-time PCR reactions were performed using the iCycler iQ detection systems (Bio-Rad). The quantitative PCR was performed according to the manufacturer's instruction (primers ID: human HIF-1α (Hs00936366_m1), RhoA (Hs00236938_m1), Rac1 (Hs01902432_s1), cdc42 (Hs00741586_mH), β-actin (FAM/MGB 4333762). mRNA expression was determined with relative quantification using threshold cycle (C_T_) values and analyzed by the 2^−ΔΔCt^. method.

### Modified Boyden chamber migration assay

The cell migration assay was performed in a modified 48-well Boyden chamber using polycarbonate nucleopore membrane with 8-μm pore size (Whatman, Piscataway, NJ, USA). Briefly, subconfluent tumor cells were trypsinized, and 20,000 cells/well were plated onto the upper chamber of each well. Fibronectin (Sigma-Aldrich) at a concentration of 100 μg/ml was added into the serum-free medium in the lower chambers. Cells were allowed to migrate for 6 hours (HT168-M1, HT1080) or 20 hours (HT25, HT29, PE/CA PJ15). Then, the upper surface of the membrane was wiped with PBS to remove any non-migrated cells. Cells that had migrated to the lower surface of the membrane were fixed with methanol, stained with Toluidine-blue solution (Sigma-Aldrich), and counted under a microscope. To measure the migration activity of cells within individual wells, the mean number of cells from five randomly chosen fields was determined at 20x magnification with an eyepiece equipped with a square grid. The results for each culture condition were expressed as the mean ± SD of six individual wells.

To avoid the effect of reoxygenation on migration capacity, HT168-M1 cells were migrated in hypoxia without 72-hour hypoxic preincubation as well.

### Time-lapse videomicroscopy

Videomicroscopy measurements were performed and analyzed as described previously [40, 41]. Briefly, the cells were plated in the inner 8 wells of 24-well plates (Sarstedt, Nümbrecht, Germany) in RPMI-1640 medium supplemented with 10% fetal bovine serum (Sigma-Aldrich). The medium was changed to CO_2_-independent medium (Gibco-BRL, Life Technologies, Rockville, MD, USA) supplemented with 10% fetal bovine serum and 4 mM glutamine after the overnight cell attachment. In order to reduce evaporation from the inner wells, the outer wells were filled with medium. Cells were kept in a custom-designed incubator built around an inverted phase-contrast microscope (World Precision Instruments, Sarasota, FL, USA) at 37°C and room ambient atmosphere. Images of 3 not overlapping microscopic view areas were taken every 5 minutes for 1 day before and 3 days after changing the oxygen tension (normoxic to hypoxic mixtures). For migration data the captured phase contrast microscope pictures were analyzed individually using a custom-made cell-tracking software enabling manual marking of individual cells. The parameter of migrated distance was calculated by averaging the displacement of the cells for each 24-hour interval for 4 consecutive days in the case of each cell line, in two independent experiments and 3 microscopic fields. The results were expressed in relative migration of the cells detected during the 72-96 h period (third day in hypoxia) relative to that observed during the first 24 hours (normoxia).

### Determination of active RhoA

Active, GTP-bound form of RhoA was determined using the G-LISA kit (Cytoskeleton, Denver, Co, USA) according to the manufacturer`s instructions.

### Western blot analysis

After incubation for 72 hours, cultured cells were washed two times by Tris-buffered saline, then lysed with MLB (Millipore, Billerica, MA, USA) cell lysis solution containing 10 μg/ml aprotinin and 10 μg/ml leupeptin, and removed from the flask surface using a cell scraper. The samples were then homogenized by pressing the lysates through 26 and 28-gauge needles (5 times) and centrifuged for 2 minutes at 14,000xg, and the supernatant was used for the analysis. The samples were diluted 1:1 by 2x Laemmli buffer (Sigma-Aldrich), and stored at −80°C until further use. The whole procedure was performed on ice to avoid protein degradation. Proteins were separated in a 10% SDS-polyacrylamide gel, then transferred to a PVDF membrane (Bio-Rad), using a wet electroblotting apparatus according to the manufacturer's protocols. Rho was detected using anti-Rho (−A, −B, −C) clone 55 (#17-294, Millipore) diluted to 3 μg/ml concentration. Rac1 and cdc42 were detected using anti-Rac1 (clone 23A8) and anti-cdc42 mouse monoclonal antibody (#17-441, Millipore) diluted to 1 μg/ml concentration. HIF-1α was detected using anti-HIF-1α rabbit monoclonal antibody (OriGene), diluted 2 μg/ml concentration. All of them were detected by HRP-conjugated goat anti-mouse IgG secondary antibody (Jackson ImmunoResearch, West Grove, PA, USA). Immunoblots were revealed by enhanced chemiluminescence system (Millipore). The bands were evaluated and quantified using GelAnalyzer 2010a software (Lazar Software, Debrecen, Hungary) and protein expressions were normalized to the level of beta-actin.

### Animal experiments

All animal-model protocols were carried out in accordance with the Guidelines for Animal Experiments and were approved by the Institutional Ethics Committee at the National Institute of Oncology, Budapest, Hungary (permission number: 22.1/722/3/2010). Male SCID mice (CB17/Icr-Prkdc*^scid^*) from our colony were used for the experiments. To analyze the effect of hypoxic conditions on metastasis formation *in vivo* we used two types of colonization and metastasis models. Single-cell suspensions were prepared from HT168-M1 monolayer cultures, washed and diluted in Medium 199 (Sigma-Aldrich). 1×10^5^ tumor cells were injected in a volume of 50 μl into the spleen of mice from where metastatic colonies formed in the liver. HT29 tumor fragments from subcutaneously growing lesions were transplanted orthotopically to the appendix region. From the latter site metastases are known to form in the liver and the lung as well. The treatments started on day 6 after tumor transplantation (10-11 mice/group were used in the case of HT168-M1, and 8 mice/group in the case of HT29). Animals on the treatment arm were randomized into three groups: control (6.25% DMSO in PBS intraperitoneally, twice a week for 4 weeks), CoCl_2_ (best known chemical inducer of hypoxia, 260 mg/l in drinking water *per os* for 4 weeks) and chetomin (Sigma-Aldrich, 1 mg/kg dissolved in DMSO intraperitoneally, twice a week for 4 weeks) groups. Chetomin is known as a selective inhibitor of HIF-1α, quenching hypoxia-inducible gene expression by inhibiting the formation of the HIF-1α/p300 complex. Experiments were terminated on day 34 after tumor inoculations. Liver and lungs with metastases were fixed in formalin and the metastases were counted under stereomicroscope.

For the investigation of HIF silencing in case of the melanoma cell line, single-cell suspensions were prepared from HT168-M1, scrambled- and shHIF-transfected monolayer cultures as well, washed and diluted in Medium 199 (Sigma). 1×10^5^ tumor cells were injected in a volume of 50 μl into the spleen of mice from where metastatic colonies formed in the liver. During the experiment, the hypoxic conditions were mimicked by CoCl_2_ treatment: 260 mg/l CoCl_2_ in the drinking water of the animals in the hypoxic arm.

### Statistical analyses

To determine statistical differences between different strata ANOVA (analyses of variance) was used with the *post hoc* Scheffé-test, where parametric methods were available. For the animal experiments we used the non-parametric Kruskal-Wallis test with *post hoc* analysis. Statistical significance was determined when *P* values were <0.05. Statistical analysis was performed by Statistica 12.0 software (StatSoft, Tulsa, OK).

## Abbreviations

ANOVA: analysis of variance
cDNA: complementary deoxyribonucleic acid
DMSO: dimethyl sulfoxide
GAP: GTPase-activating protein
GDI: guanosine nucleotide dissociation inhibitor
GEF: Guanine nucleotide exchange factors
HIF-1α: Hypoxia-inducible factor 1-alpha
HIF-1β: Hypoxia-inducible factor 1-beta
HRE: Hypoxia-Response Element
HRP: horseradish peroxidase
i.p.: intraperitoneal
mRNA: messenger ribonucleic acid
PBS: Phosphate-buffered saline
PCR: Polymerase chain reaction
PVDF: Polyvinylidene fluoride
SCID: Severe combined immunodeficiency
SD: standard deviation
SEM: standard error of mean
shRNA: short hairpin ribonucleic acid
SRB: sulforhodamine B
VEGF: Vascular endothelial growth factor
